# Sex Differences in Percutaneous Coronary Intervention—Insights From the Coronary Angiography and PCI Registry of the German Society of Cardiology

**DOI:** 10.1161/JAHA.116.004972

**Published:** 2017-03-20

**Authors:** Tobias Heer, Matthias Hochadel, Karin Schmidt, Julinda Mehilli, Ralf Zahn, Karl‐Heinz Kuck, Christian Hamm, Michael Böhm, Georg Ertl, Hans Martin Hoffmeister, Stefan Sack, Jochen Senges, Steffen Massberg, Anselm K. Gitt, Uwe Zeymer

**Affiliations:** ^1^ Klinikum München Schwabing Academic Teaching Hospital University of Munich Germany; ^2^ Stiftung Institut für Herzinfarktforschung Ludwigshafen Ludwigshafen Germany; ^3^ Medizinische Klinik B Klinikum der Stadt Ludwigshafen Ludwigshafen Germany; ^4^ Medizinische Klinik I Klinikum der Ludwig‐Maximilians‐Universität München Munich Germany; ^5^ Asklepios Klinik St. Georg Hamburg Hamburg Germany; ^6^ Kerckhoff Klinik Bad Nauheim Bad Nauheim Germany; ^7^ Universitätsklinikum des Saarlandes Homburg/Saar Germany; ^8^ Medizinische Klinik und Poliklinik I/Comprehensive Heart Failure Center Universitätsklinikum Würzburg Würzburg Germany; ^9^ Städtisches Klinikum Solingen Solingen Germany

**Keywords:** acute coronary syndrome, angioplasty and stenting, coronary artery disease, outcome, women, Cardiovascular Disease, Women, Percutaneous Coronary Intervention, Stent, Treatment

## Abstract

**Background:**

Several studies have suggested sex‐related differences in diagnostic and invasive therapeutic coronary procedures.

**Methods and Results:**

Data from consecutive patients who were enrolled in the Coronary Angiography and PCI Registry of the German Society of Cardiology were analyzed. We aimed to compare sex‐related differences in in‐hospital outcomes of patients undergoing percutaneous coronary intervention (PCI) for stable coronary artery disease, non‐ST elevation acute coronary syndromes, ST elevation myocardial infarction, and cardiogenic shock. From 2007 until the end of 2009 data from 185 312 PCIs were prospectively registered: 27.9% of the PCIs were performed in women. Primary PCI success rate was identical between the sexes (94%). There were no sex‐related differences in hospital mortality among patients undergoing PCI for stable coronary artery disease, non‐ST elevation acute coronary syndromes, or cardiogenic shock except among ST elevation myocardial infarction patients. Compared to men, women undergoing primary PCI for ST elevation myocardial infarction have a higher risk of in‐hospital death, age‐adjusted odds ratio (1.19, 95% CI 1.06‐1.33), and risk of ischemic cardiac and cerebrovascular events (death, myocardial infarction, transient ischemic attack/stroke), (age‐adjusted odds ratio 1.19, 95% CI 1.16‐1.29). Furthermore, access‐related complications were twice as high in women, irrespective of the indication.

**Conclusions:**

Despite identical technical success rates of PCI between the 2 sexes, women with PCI for ST elevation myocardial infarction have a 20% higher age‐adjusted risk of death and of ischemic cardiac and cerebrovascular events. Further research is needed to determine the reasons for these differences.

## Introduction

Recently, the German Heart Report 2013 described a decrease in total mortality rate of acute myocardial infarction in men since 1980 and in women since 1995. In men, there was a decrease of about 50%, and in women of about 35%.^1^ This is in line with data from the World Health Organization global mortality database, which describe a decrease in age‐standardized coronary heart disease mortality in the last 25 years of 49% in men and 39% in women.^2^ There has been speculation about possible explanations for these sex differences, such as delay in seeking medical care for acute coronary syndromes with longer time from symptom onset to balloon in women, sex bias in use of evidence‐based and guideline‐recommended lifesaving medical therapy, differences in use of drug‐eluting stents, thrombolysis, emergency percutaneous coronary intervention (PCI), or timely coronary artery bypass graft (CABG) procedures.^1^, ^2^ In addition, it has been reported that only an estimated 33% of annual PCIs were performed in women despite the established benefits of PCI in reducing fatal and nonfatal ischemic complications in patients with acute coronary syndromes.^3^ It has been found that women were less likely to be revascularized than men, either in the management of stable angina^4^ or in acute coronary syndromes.^5^, ^6^ Recently, we have been able to show that in patients with significant coronary artery disease there was no difference in recommendation for PCI between women and men.^7^ Nearly all of the studies found a higher crude hospital mortality in women with PCI for acute coronary syndromes. In some of the studies the difference in hospital mortality between the sexes disappeared after adjustment for age and comorbidity,^5^, ^6^, ^8^, ^9^, ^10^, ^11^ whereas in others it persisted.^12^, ^13^, ^14^, ^15^, ^16^, ^17^ Interestingly, despite the pronounced decrease in mortality for men, the long‐term prognosis after an acute myocardial infarction was still less favorable in men than in women in Germany and in all other European countries.^1^, ^2^

The purpose of this study was to examine sex differences in procedural features, safety, and efficacy of PCI in a very large coronary angiography and PCI registry in Germany.

## Methods

The data presented in this analysis were taken from 218 hospitals that transferred data to the Bundesgeschäftsstelle Qualitätssicherung gGmbH (BQS) or enrolled patients in the German Arbeitsgemeinschaft Leitende Kardiologische Krankenhausärzte (ALKK) registry. The comprehensive collection and analysis of these data was endorsed by the German Cardiac Society. Details of the BQS quality program and the ALKK registry have been described before.^7^, ^18^, ^19^, ^20^ Since 2002 all hospitals that perform invasive coronary procedures in Germany were legally obligated to record details about these procedures to monitor quality control. Therefore, consent of patients for the processing of their anonymized data was generally obtained. The data contain all consecutive PCIs of the participating hospitals on an intention‐to‐treat basis. Baseline characteristics, course of PCI, and hospital complications were registered prospectively and analyzed centrally at the Karl Ludwig Neuhaus Datenzentrum, Stiftung Institut für Herzinfarktforschung, Ludwigshafen, Germany. All information about success of PCI is based on the evaluation of the treating physician. PCI was rated successful if there was a residual stenosis of <50% after balloon angioplasty without stenting and <20% after PCI with stent application. The present analysis comprises all patients who were treated with PCI for stable coronary artery disease, non‐ST elevation acute coronary syndromes (NSTE‐ACS), and ST elevation myocardial infarction (STEMI). The first PCI performed in each patient during hospital stay was considered for the analysis.

### Data Collection

Data on patient characteristics on admission comprised age, sex, concomitant diseases, cardiovascular risk factors, prior PCI or CABG, prior myocardial infarction, or stroke. Following completion of the procedure, lesion characteristics, details of the intervention, and intraprocedural and in‐hospital adverse cardiovascular outcomes were recorded on a standardized form by the treating physician and transmitted to the central data registry center in Ludwigshafen via the Internet by the treating physician.

### Definitions

STEMI was defined as ST‐segment elevation of ≥2 mm in ≥2 precordial leads or >1 mm in ≥2 extremity leads; non‐ST elevation myocardial infarction (NSTEMI) was defined as any significant increase in troponin level without ST elevation in the defining ECG. Adverse outcomes (major adverse cardiac or cerebrovascular events), defined as in‐hospital death, myocardial infarction, stroke, or reinfarction, were evaluated. Myocardial infarction was defined as a 2‐fold rise in creatine kinase level above normal. A cerebrovascular event was defined as a stroke or a transitory or reversible cerebral ischemic attack that was confirmed clinically as a permanent neurological deficit or by CT scan. In addition, pulmonary embolism, resuscitation, complications at the puncture site, acute renal failure, requirement for dialysis, septicemia, and urgent CABG procedure were reported as in‐hospital events.

Primary success of PCI was defined as the achievement of <20% final diameter stenosis after stent implantation and <50% after coronary angioplasty without stent implantation in visual assessment associated with Thrombolysis In Myocardial Infarction (TIMI) flow grade ≥2.

### Statistical Analysis

Statistical analysis was performed using the SAS software package version 9.3 (SAS Inc, Cary, NC). Binary data are expressed as percentages (numerator/denominator numbers); for continuous variables such as age and hospital stay, median values with 25th and 75th percentiles (interquartile range) are shown. The descriptive statistics are based on the available cases, which are used as denominator of rates. The Pearson χ^2^ test was used to examine differences between patient groups in categorical variables, and the Mann‐Whitney‐Wilcoxon test for metrically scaled variables. Statistical significance was defined as 2‐sided *P*≤0.05. Odds ratios (OR) with 95% CIs were calculated for the comparison of binary variables. OR with 95% CIs adjusted for age as a linear term were calculated by logistic regression for sex comparisons.

## Results

From 2007 until the end of 2009, data of 185 312 PCIs from a total of 218 German hospitals were prospectively registered. Sex differences in therapeutic recommendation after diagnostic coronary angiography were described before.^7^

### Sex Differences in Baseline and Procedural Characteristics, All Procedures

Of patients undergoing PCI, 27.9% were female. Women were 5 years older than men (72 years vs 67 years, *P*<0.001). Compared to men, they less often had a history of former coronary angiography (44.6% vs 52.9%, OR 0.72, 95% CI 0.70‐0.73), of PCI (33.2% vs 40.7%, OR 0.72, 95% CI 0.71‐0.74), and of CABG (8.4% vs 13.7%, OR 0.58, 95% CI 0.56‐0.60). Women more often had diabetes mellitus (29.5% vs 23.7%, OR 1.34, 95% CI 1.31‐1.38), and they more often presented with congestive heart failure (8.5% vs 7.8%, OR 1.09, 95% CI 1.05‐1.13). Procedural success was slightly higher in women than in men (94.5% vs 94%, OR 1.09, 95% CI 1.04‐1.14), but procedural complications were higher in women, including death in the catheterization laboratory (0.3% vs 0.2%, OR 1.47, 95% CI 1.21‐1.80) and resuscitation (0.8% vs 0.5%, OR 1.43, 95% CI 1.27‐1.62). The absolute number of TIAs/strokes was 23/51 771 in women and 33/133 530 in men (OR 1.80, 95% CI 1.06‐3.06).

### Sex Differences in Baseline and Procedural Characteristics According to the Indication for First PCI

Results are shown in Tables 1 and S1. In elective PCI the primary success rate was higher in women compared to men, the stent rate was higher, as was the number of PCI in ostial stenoses. In contrast, men more often had PCI in more than 1 vessel, more often with complete vessel occlusion, more often in bypass grafts, more often in an unprotected left main stenosis, and more often in the last coronary vessel. There was no difference in acute mortality in the catheterization laboratory between the 2 sexes (see Tables 1 and S1).

**Table 1:** Baseline and Procedural Characteristics

	Women	Men	Age‐Adjusted OR (95% CI)
PCI in elective patients			
	n=24 262 (26.9%)	n=65 972 (73.1%)	
Age, y	72 (65; 78)	68 (60; 74)	
History of PCI	42.9% (10 316/24 066)	51.6% (33 711/65 308)	0.72 (0.69‐0.74)
History of CABG surgery	10.1% (2443/24 178)	16.5% (10 811/65 684)	0.50 (0.47‐0.52)
LV ejection fraction ≤40%	8.9% (1946/21 805)	13.4% (7921/59 019)	0.59 (0.56‐0.62)
Diabetes mellitus	29.8% (7124/23 879)	25.0% (16 222/64 800)	1.20 (1.16‐1.24)
Renal insufficiency	16.5% (3931/23 862)	16.7% (10 863/64 874)	0.79 (0.76‐0.82)
Diagnostic XA and PCI in 1 session	84.8% (20 565/24 262)	83.9% (55 371/65 972)	1.08 (1.04‐1.13)
PCI successful^a^	95.2% (23 093/24 262)	94.1% (62 051/65 972)	1.25 (1.16‐1.33)
Stent implanted	90.0% (21 829/24 262)	88.9% (58 651/65 972)	1.11 (1.06‐1.17)
PCI in more than 1 vessel	8.9% (2169/24 261)	11.0% (7224/65 972)	0.78 (0.74‐0.82)
PCI in complete vessel occlusion	7.5% (1808/24 181)	9.2% (6028/65 733)	0.87 (0.82‐0.92)
PCI in CABG	2.1% (512/24 181)	4.6% (3024/65 733)	0.38 (0.35‐0.42)
PCI in unprotected left main	1.2% (288/24 181)	1.4% (935/65 733)	0.76 (0.67‐0.87)
PCI in last coronary vessel	0.3% (67/24 181)	0.4% (233/65 733)	0.72 (0.55‐0.95)
PCI in NSTE‐ACS patients without cardiogenic shock			
	n=14 336 (29.7%)	n=33 879 (70.3%)	
Age, y	74 (66; 80)	68 (58; 75)	
History of PCI	30.0% (4227/14 101)	35.6% (11 872/33 326)	0.72 (0.69‐0.75)
History of CABG surgery	8.9% (1263/14 234)	15.0% (5043/33 623)	0.44 (0.41‐0.47)
LV ejection fraction ≤40%	13.6% (1561/11 437)	16.8% (4525/26 886)	0.66 (0.62‐0.70)
Diabetes mellitus	31.6% (4367/13 808)	25.1% (8205/32 649)	1.22 (1.17‐1.28)
Renal insufficiency	21.5% (2989/13 888)	20.7% (6807/32 806)	0.73 (0.70‐0.77)
On hemodialysis	1.9% (257/13 888)	2.0% (643/32 806)	0.83 (0.72‐0.97)
Diagnostic XA and PCI in 1 session	95.3% (13 662/14 336)	95.1% (32 230/33 879)	1.14 (1.04‐1.25)
PCI successful^a^	94.6% (13 556/14 336)	94.1% (31 893/33 878)	1.17 (1.07‐1.28)
Stent implanted	90.2% (12 935/14 336)	89.8% (30 436/33 879)	1.10 (1.02‐1.17)
PCI in more than 1 vessel	11.9% (1703/14 336)	11.8% (3987/33 879)	0.93 (0.88‐0.99)
PCI in complete vessel occlusion	17.4% (2486/14 288)	20.3% (6836/33 753)	0.92 (0.88‐0.97)
PCI in CABG	3.0% (429/14 288)	6.8% (2301/33 753)	0.33 (0.30‐0.37)
PCI in ostial stenosis	7.0% (1007/14 288)	5.7% (1910/33 753)	1.13 (1.04‐1.22)
Resuscitation in cath lab	0.6% (88/14 335)	0.3% (101/33 877)	1.78 (1.32‐2.39)
PCI in STEMI‐ACS patients without cardiogenic shock			
	n=9156 (27.8%)	n=23 830 (72.2%)	
Age, y	72 (61; 79)	62 (53; 71)	
History of PCI	12.4% (1103/8875)	17.6% (4057/23 112)	0.59 (0.55‐0.64)
History of CABG surgery	2.8% (251/9028)	4.5% (1065/23 562)	0.42 (0.37‐0.49)
LV ejection fraction ≤40%	19.7% (1161/5894)	18.6% (2849/15 304)	0.92 (0.85‐0.99)
Diabetes mellitus	25.5% (2115/8282)	17.7% (3811/21 547)	1.31 (1.23‐1.40)
Renal insufficiency	16.4% (1305/7934)	12.3% (2550/20 690)	0.87 (0.81‐0.94)
On hemodialysis	98.1% (8978/9156)	98.1% (23 372/23 830)	1.08 (0.90‐1.30)
Diagnostic XA and PCI in 1 session	93.5% (8557/9156)	94.7% (22 568/23 830)	0.96 (0.87‐1.07)
PCI successful^a^	91.1% (8341/9156)	92.4% (22 028/23 830)	0.96 (0.87‐1.05)
Stent implanted	1.1% (103/9146)	2.2% (517/23 818)	0.33 (0.27‐0.42)
PCI in more than 1 vessel	0.1% (5/9156)	<0.1% (3/23 830)	4.94 (1.12‐21.72)
PCI in complete vessel occlusion	1.4% (127/9156)	1.0% (231/23 830)	1.32 (1.06‐1.66)
PCI in CABG	12.4% (1103/8875)	17.6% (4057/23 112)	0.59 (0.55‐0.64)
PCI in ostial stenosis	2.8% (251/9028)	4.5% (1065/23 562)	0.42 (0.37‐0.49)
Resuscitation in cath lab	19.7% (1161/5894)	18.6% (2849/15 304)	0.92 (0.85‐0.99)
PCI in cardiogenic shock			
	n=963 (30.3%)	n=2219 (69.7%)	
Age, y	74 (66; 81)	67 (57; 75)	
History of PCI	17.8% (154/864)	25.6% (498/1943)	0.59 (0.48‐0.72)
History of CABG surgery	7.0% (64/919)	10.2% (218/2145)	0.55 (0.41‐0.75)
LV ejection fraction ≤40%	62.4% (406/651)	66.9% (984/1471)	0.77 (0.63‐0.94)
Diabetes mellitus	35.4% (274/775)	31.4% (544/1735)	1.00 (0.83‐1.20)
Renal insufficiency	37.5% (291/776)	36.6% (654/1788)	0.76 (0.63‐0.92)
STEMI	77.6% (747/963)	73.7% (16 536/2219)	1.45 (1.21‐1.75)
Diagnostic XA and PCI in 1 session	97.9% (943/963)	98.0% (2175/2219)	0.99 (0.57‐1.72)
PCI successful^a^	86.1% (829/963)	85.7% (1901/2219)	1.19 (0.95‐1.49)
Stent implanted	86.1% (829/963)	85.9% (1907/2219)	1.13 (0.90‐1.41)
PCI in complete vessel occlusion	65.3% (628/962)	62.0% (1376/2218)	1.29 (1.09‐1.51)
PCI in CABG	2.5% (24/962)	4.7% (105/2218)	0.41 (0.26‐0.64)
PCI in last coronary vessel	2.8% (27/962)	4.3% (96/2218)	0.62 (0.39‐0.96)

Values are expressed as percentages (number of occurrences/available cases) except for age (median; interquartile range). Only relevant data are shown. The complete tables can be found in Tables S1 through S4. CABG indicates coronary artery bypass graft; LV, left ventricle; NSTE‐ACS, non‐ST elevation acute coronary syndrome; OR, odds ratio; PCI, percutaneous coronary intervention; STEMI, ST elevation myocardial infarction; XA, coronary angiography.

a Defined as <20% final diameter stenosis after stent implantation and <50% after coronary angioplasty without stent implantation in visual assessment associated with TIMI flow grade ≥2.

Sex differences in NSTE‐ACS were comparable to that of elective PCI. Primary success rate and stent rate were higher in women. PCI in more than 1 area, PCI in complete vessel occlusion, PCI in CABG, PCI in an unprotected left main, and PCI in last coronary vessel had higher incidence in men than in women, but PCI in ostial stenosis was more frequent in women (see Tables 1 and S2).

In STEMI, sex differences in procedural characteristics were less pronounced. There was no difference in success rate between men and women. Resuscitation in the catheterization laboratory was more often necessary in women, and PCI in bypass grafts was performed more frequently in men than in women (see Tables 1 and S3).

In cardiogenic shock, there was no sex difference in primary success rate between the sexes. PCI in CABG was less often performed in women. Resuscitation in the catheterization laboratory was more often necessary in women than in men (see Tables 1 and S4).

### Sex Differences in In‐Hospital Course and Procedure‐Related Complications According to the Indication for First PCI

**Figure 1:**
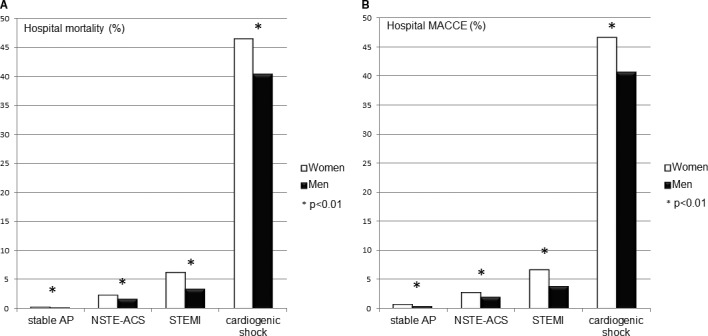
Hospital mortality (A) and hospital MACCE (B) for women and men after elective percutaneous coronary intervention for stable angina pectoris (AP), non‐ST elevation acute coronary syndrome (NSTE‐ACS), ST elevation myocardial infarction (STEMI), and cardiogenic shock in the univariate analysis (MACCE [major adverse cardiac or cerebrovascular event]=death, nonfatal myocardial infarction, hospital transient ischemic attack, and hospital stroke).

Results are shown in Table 2, Tables S5 through S8, and Figure 1A and 1B. After adjustment for age, hospital mortality after PCI was higher in women with PCI in STEMI but not in elective PCI, PCI in NSTE‐ACS, or PCI in cardiogenic shock. Hospital major adverse cardiac or cerebrovascular events were higher in women with elective PCI and in PCI for STEMI. Unadjusted access‐related complications were twice as high in women in PCI for all indications.

**Table 2:** In‐Hospital Outcomes

	Women	Men	Age‐Adjusted OR (95% CI)
PCI in elective patients			
	n=24 262 (26.9%)	n=65 972 (73.1%)	
Hospital death	0.3% (82/24 262)	0.2% (154/65 972)	1.07 (0.82‐1.41)
Hospital MACE	0.7% (162/24 262)	0.4% (272/65 972)	1.37 (1.12‐1.67)
Hospital MACCE	0.8% (184/24 262)	0.5% (323/65 972)	1.31 (1.09‐1.57)
Nonfatal resuscitation	0.2% (44/24 262)	0.1% (90/65 972)	1.33 (0.92‐1.91)
Access‐related complications	1.8% (447/24 245)	0.8% (553/65 932)	2.07 (1.83‐2.36)
PCI in NSTE‐ACS patients without cardiogenic shock			
	n=14 336 (29.7%)	n=33 879 (70.3%)	
Hospital death	2.4% (350/14 336)	1.8% (597/33 879)	1.02 (0.89‐1.16)
Hospital MACE	2.7% (388/14 336)	2.0% (670/33 879)	1.04 (0.91‐1.18)
Hospital MACCE	2.9% (415/14 336)	2.1% (704/33 879)	1.06 (0.94‐1.21)
Nonfatal resuscitation	0.3% (47/14 336)	0.2% (67/33 879)	1.61 (1.10‐2.37)
Access‐related complications	2.3% (324/14 299)	1.0% (352/33 844)	1.99 (1.70‐2.33)
PCI in STEMI‐ACS patients without cardiogenic shock			
	n=9156 (27.8%)	n=23 830 (72.2%)	
Hospital death	6.3% (581/9156)	3.6% (854/23 830)	1.19 (1.06‐1.33)
Hospital MACE	6.6% (608/9156)	3.8% (906/23 830)	1.19 (1.07‐1.34)
Hospital MACCE	6.8% (621/9156)	3.9% (924/23 830)	1.20 (1.08‐1.34)
Nonfatal resuscitation	0.7% (65/9156)	0.6% (154/23 830)	1.18 (0.87‐1.59)
Access‐related complications	2.2% (201/9142)	0.8% (201/23 808)	2.32 (1.89‐2.85)
PCI in cardiogenic shock			
	n=963 (30.3%)	n=2219 (69.7%)	
Hospital death	46.5% (448/963)	40.4% (896/2219)	1.05 (0.89‐1.23)
Hospital MACE	46.6% (449/963)	40.6% (902/2219)	1.04 (0.89‐1.22)
Hospital MACCE	46.6% (449/963)	40.7% (903/2219)	1.04 (0.89‐1.22)
Nonfatal resuscitation	2.7% (26/963)	3.2% (70/2219)	0.93 (0.59‐1.49)
Access‐related complications	1.1% (11/961)	0.4% (9/2206)	2.66 (1.07‐6.63)

Values are expressed as percentages (number of occurrences/available cases). Only relevant data are shown. The complete tables can be found in Tables S5 through S8. MACCE, major adverse cardiac or cerebrovascular event (death, nonfatal myocardial infarction, hospital transient ischemic attack, and hospital stroke); MACE, major adverse cardiac event (death, nonfatal myocardial infarction); NSTE‐ACS, non‐ST elevation acute coronary syndrome; OR, odds ratio; PCI, percutaneous coronary intervention; STEMI, ST elevation myocardial infarction.

### Sex Differences in Hospital Mortality in Different Age Groups

**Figure 2:**
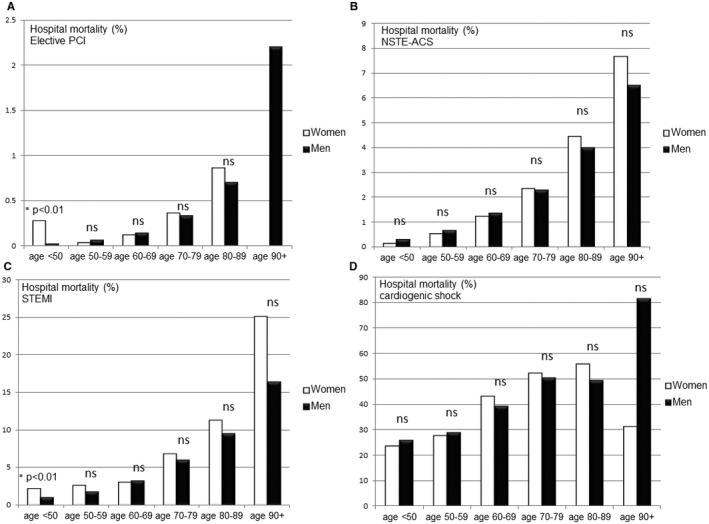
Sex differences in hospital mortality after elective percutaneous coronary intervention (PCI) (A), non‐ST elevation acute coronary syndrome (NSTE‐ACS) (B), ST elevation myocardial infarction (STEMI) (C), and cardiogenic shock (D) in different age groups in a univariate analysis.

Hospital mortality in women <50 years of age with elective PCI or with PCI for STEMI was significantly higher compared to men in the same age group. Differences in the other age groups were not significant (Figure 2A through 2D).

### Sex Differences in Predictors for Hospital Mortality in STEMI, NSTE‐ACS, and Elective PCI, Multivariate Analysis

**Figure 3:**
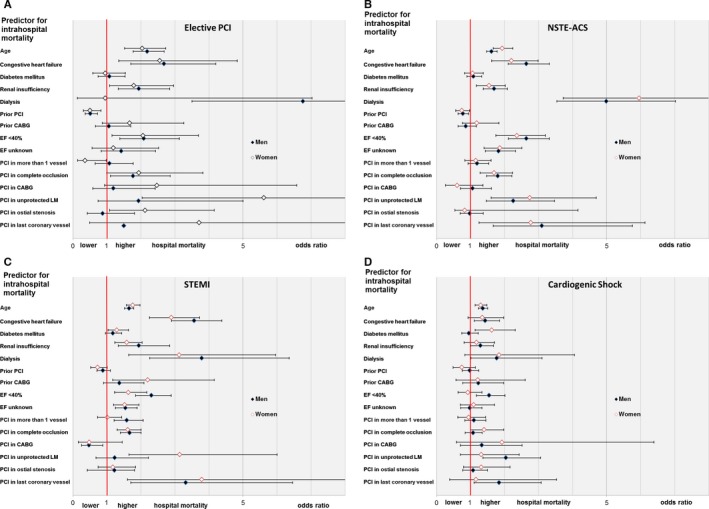
Sex differences in predictors for intrahospital mortality, elective percutaneous coronary intervention (A), non‐ST elevation acute coronary syndrome (NSTE‐ACS) (B), ST elevation myocardial infarction (STEMI) (C), and cardiogenic shock (D). The figure shows odds ratios with 95% confidence intervals. CABG indicates coronary artery bypass graft; EF, ejection fraction; LM, left main coronary artery; PCI, percutaneous coronary intervention.

In a multivariate analysis for predictors of hospital mortality, we found different predictors that were associated with increased hospital mortality in stable CAD, NSTE‐ACS, STEMI, and cardiogenic shock (see Figure 3).

## Discussion

The present analysis gives a representative overview of sex‐associated differences in PCI in Germany from 2007 until 2009. We found that 27.8% of all PCIs were performed in women despite the fact that mortality rates due to coronary heart disease in Germany are almost the same in women and men.^1^, ^2^ This rate of PCI is comparable to that in the United States.^3^ It is not yet completely understood why this difference in use of invasive coronary procedures between men and women exists in spite of the established benefits of PCI in reducing fatal and nonfatal ischemic complications.^21^

Women with PCI were older than men, and they had more comorbidity. The largest difference of about 10 years was found in STEMI patients, which has been described before.^5^ We did not find any evidence that primary success rate of PCI was lower in women than in men. In elective PCI and NSTE‐ACS, it was even higher in women than in men, as was the percentage of implanted stents. This finding is in line with former reports in which it was found that the technical success of PCI is similar in women and in men.^11^

Procedural and postinterventional hospital complications were significantly higher in women compared to men, even after adjustment for age. This has been described before,^5^, ^6^, ^11^, ^22^, ^23^ but the reasons for this finding are speculative (smaller vessel size in women, higher comorbidity, differences in endothelial function, variability in correct dosage of adjunctive medical therapy). Although the overall superiority of primary PCI in acute myocardial infarction over fibrinolytic therapy has been clearly demonstrated for women,^3^, ^10^ in FRISC II and RITA‐3 an early invasive strategy in NSTE‐ACS did not reduce the risk of future events among women with unstable angina or NSTEMI.^24^, ^25^ In other studies women with PCI for acute coronary syndromes showed a lower or at least identical long‐term mortality compared to men.^5^, ^6^, ^11^, ^23^ Hospital mortality after PCI for STEMI in our population was higher in women compared to men—even after adjustment for age and other covariables. Yet, it has considerably decreased in the last decade in men and women, both in STEMI and NSTE‐ACS.^2^, ^3^, ^5^, ^6^, ^21^, ^26^, ^27^, ^28^, ^29^, ^30^ In line with former studies we found that younger women with STEMI have higher hospital mortality.^2^, ^5^, ^31^ In comparison to historical data from the MITRA registry (data from 1994 to 1997, hospital mortality in women 20.9%, in men 12.3%), hospital mortality after STEMI has dramatically decreased in both sexes, but there is still a sex difference with a 20% higher mortality in women after adjustment for age. Not unexpectedly, hospital mortality was lowest in elective PCI and highest in cardiogenic shock without differences between the sexes after age‐adjustment.

Interestingly, sex differences in vascular complications after PCI were comparable to that after lone diagnostic coronary angiography, and they were lower compared to former reports, which may reflect the decreasing use of aggressive anticoagulation regimes, the use of weight‐adjusted heparin dosing, and the introduction of smaller sheath sizes and early sheath removal.^3^

As for subgroup analysis, we did not find any relevant sex differences in specific risk groups. Regarding procedure related differences, we found that PCI in more than 1 coronary vessel (non‐culprit PCI) in STEMI was associated with a higher mortality in men, but not in women, and not in NSTE‐ACS. In patients without acute coronary syndromes, PCI in more than 1 vessel, PCI in CABG, and PCI in unprotected left main coronary artery were associated with higher mortality in women, but not in men. The reasons for these procedure‐associated differences in hospital mortality cannot be explained by the data of this registry and should be further investigated.

### Limitations

Our data are taken from a German population and describe the treatment reality in German hospitals. So our data may be not generalizable to other populations in other countries. As we report about an invasive coronary angiography/PCI registry we have no data about patients who were primarily treated noninvasively. The diagnosis of CAD is subjective and not based on objective measurement (such as quantitative coronary angiography). We have no information about the vascular access, but we guess that the rate of radial access in the years 2007‐2009 was less than 10% in Germany. The completeness of revascularization in both sexes cannot be evaluated with our data. The present analysis is not a randomized, controlled study, and all treatment decisions were left to the discretion of the physician. There was no documentation of concomitant medical treatment. The analysis was designed after the data had been collected. Therefore, the results have to be interpreted as descriptive. On the other hand, this investigation is one the largest analyses on a contemporary population and, therefore, is likely to reliably reflect the current clinical practice.

## Conclusion

The present analysis gives a representative overview of sex‐associated differences in PCI for different indications in Germany. Hospital mortality after PCI for STEMI was 20% higher in women than in men, but there was no difference in hospital mortality after PCI for NSTE‐ACS, for cardiogenic shock, and after elective PCI. Nonfatal postinterventional major adverse cardiac and cerebrovascular events were higher in women with PCI for STEMI and after elective PCI. Access‐related complications were twice as high in women as in men, irrespective of the indication. The reasons for these differences need to be addressed in future research.

## Sources of Funding

This work was supported by the German Society of Cardiology and The Stiftung Institut für Herzinfarktforschung Ludwigshafen. The sponsor of the study had no role in study design, data collection, data analysis, data interpretation, or writing of the report.

## Disclosures

None.

## Supporting information


**Table S1.** Baseline and Procedural Characteristics, Elective PCI
**Table S2.** Baseline and Procedural Characteristics, PCI in NSTE‐ACS, No Cardiogenic Shock
**Table S3.** Baseline and Procedural Characteristics, PCI in STEMI, No Cardiogenic Shock
**Table S4.** Baseline and Procedural Characteristics, PCI in Cardiogenic Shock
**Table S5.** In‐Hospital Course and Procedure‐Related Complications, Elective PCI
**Table S6.** Hospital Course and Procedure‐Related Complications, PCI in NSTE‐ACS, No Cardiogenic Shock
**Table S7.** Hospital Course and Procedure‐Related Complications, PCI in STEMI, No Cardiogenic Shock
**Table S8.** Hospital Course and Procedure‐Related Complications, PCI in Cardiogenic Shock
