# Supplementary material for: Sex Differences in Percutaneous Coronary Intervention—Insights From the Coronary Angiography and PCI Registry of the German Society of Cardiology
Source: J Am Heart Assoc. 2017 Mar 20;6(3):e004972. doi: 10.1161/JAHA.116.004972 (PMC5524024; doi:10.1161/JAHA.116.004972)
Supplement: Supplementary file 1 — Table S1. Baseline and Procedural Characteristics, Elective PCI Table S2. Baseline and Procedural Characteristics, PCI in NSTE‐ACS, No Cardiogenic Shock Table S3. Baseline and Procedural Characteristics, PCI in STEMI, No Cardiogenic Shock Table S4. Baseline and Procedural Characteristics, PCI in Cardiogenic Shock Table S5. In‐Hospital Course and Procedure‐Related Complications, Elective PCI Table S6. Hospital Course and Procedure‐Related Complications, PCI in NSTE‐ACS, No Cardiogenic Shock Table S7. Hospital Course and Procedure‐Related Complications, PCI in STEMI, No Cardiogenic Shock Table S8. Hospital Course and Procedure‐Related Complications, PCI in Cardiogenic Shock [file JAH3-6-e004972-s001.pdf]

SUPPLEMENTAL MATERIAL

Table S1. Base-line and procedural characteristics, elective PCI

	Women<br>n= 24262 (26,9 %)	Men<br>n= 65972 (73,1 %)	Odds ratio<br>(95%-CI)	Age-adjusted<br>OR (95%-CI)
Age (years)*	72 (65; 78)	68 (60; 74)		
Prior XA	57.2 % (13803/24124)	66.4 % (43508/65519)	0.68 (0.66-0.70)	0.66 (0,64-0.68)
Prior PCI	42.9 % (10316/24066)	51.6 % (33711/65308)	0.70 (0.68-0.72)	0.72 (0.69-0.74)
Prior CABG	10.1 % (2443/24178)	16.5 % (10811/65684)	0.57 (0.54-0.60)	0.50 (0.47-0.52)
Ejection fraction ≤40%	8.9 % (1946/21805)	13.4 % (7921/59019)	0.63 (0.60-0.67)	0.59 (0.56-0.62)
Diabetes mellitus	29.8 % (7124/23879)	25.0 % (16222/64800)	1.27 (1.23-1.32)	1.20 (1.16-1.24)
Renal insufficiency	16.5 % (3931/23862)	16.7 % (10863/64874)	0.98 (0.94-1.02)	0.79 (0.76-0.82)
On hemodialysis	1.3 % (310/23862)	1.2 % (768/64874)	1.10 (0.96-1.25)	1.06 (0.93-1.21)
Cardiac dyspnoea	57.1 % (13859/24261)	50.2 % (33106/65972)	1.32 (1.28-1.36)	1.22 (1.18-1.25)
Other symptoms (e.g. arrhythmia)	25.5 % (6196/24261)	25.7 % (16952/65972)	0.99 (0.96-1.03)	0.93 (0.89-0.96)
Diagnostic XA and PCI in one session	84.8 % (20565/24262)	83.9 % (55371/65972)	1.06 (1.02-1.11)	1.08 (1.04-1.13)
- fluoroscopy time (min)*	8.3 (5.3; 13.4)	9.0 (5.8; 14.8)		
- contrast agent (ml)*	170 (130; 230)	180 (140; 240)		
PCI only	15.2 % (3697/24262)	16.1 % (10601/65972)	0.94 (0.90-0.98)	0.93 (0.89-0.96)
- fluoroscopy time (min)*	7.3 (4.5; 13.0)	8.3 (5.0; 14.5)		
- contrast agent (ml)*	120 (90; 180)	140 (100; 200)		
PCI successful	95.2 % (23093/24262)	94.1 % (62051/65972)	1.25 (1.17-1.33)	1.25 (1.16-1.33)
Stent implanted	90.0 % (21829/24262)	88.9 % (58651/65972)	1.12 (1.07-1.18)	1.11 (1.06-1.17)
PCI in more than 1 vessel	8.9 % (2169/24261)	11.0 % (7224/65972)	0.80 (0.76-0.84)	0.78 (0.74-0.82)
PCI in complete vessel occlusion	7.5 % (1808/24181)	9.2 % (6028/65733)	0.80 (0.76-0.85)	0.87 (0.82-0.92)
PCI in CABG	2.1 % (512/24181)	4.6 % (3024/65733)	0.45 (0.41-0.49)	0.38 (0.35-0.42)
PCI in unprotected left main	1.2 % (288/24181)	1.4 % (935/65733)	0.84 (0.73-0.95)	0.76 (0.67-0.87)
PCI in ostial stenosis	6.0 % (1440/24181)	5.2 % (3407/65733)	1.16 (1.09-1.23)	1.11 (1.04-1.18)
PCI in last coronary vessel	0.3 % (67/24181)	0.4 % (233/65733)	0.78 (0.60-1.03)	0.72 (0.55-0.95)
Death in cath lab	<0.1 % (6/24261)	<0.1 % (6/65970)	2.72 (0.88-8.43)	2.49 (0.79-7.90)
TIA/stroke in cath lab	<0.1 % (6/24261)	<0.1 % (12/65970)	1.36 (0.51-3.62)	1.15 (0.42-3.10)
Resuscitation in cath lab	0.2 % (39/24261)	0.1 % (89/65970)	1.19 (0.82-1.74)	1.17 (0.80-1.72)

Values are expressed as percentages (number of occurrences/available cases) unless indicated otherwise

*Median (interquartile range).

PCI=percutaneous coronary intervention, OR=odds ratio, CI=confidence interval, XA=coronary angiography, CABG=coronary artery bypass graft

Table S2. Base-line and procedural characteristics, PCI in NSTE-ACS, no cardio-genic shock

	Women<br>n= 14336 (29,7 %)	Men<br>n= 33879 (70,3 %)	Odds ratio<br>(95%-CI)	Age-adjusted<br>OR (95%-CI)
Age (years)*	74 (66; 80)	68 (58; 75)		
Prior XA	40.9 % (5790/14155)	47.9 % (16019/33462)	0.75 (0.72-0.78)	0.66 (0.63-0.69)
Prior PCI	30.0 % (4227/14101)	35.6 % (11872/33326)	0.77 (0.74-0.81)	0.72 (0.69-0.75)
Prior CABG	8.9 % (1263/14234)	15.0 % (5043/33623)	0.55 (0.52-0.59)	0.44 (0.41-0.47)
Ejection fraction ≤40%	13.6 % (1561/11437)	16.8 % (4525/26886)	0.78 (0.73-0.83)	0.66 (0.62-0.70)
Diabetes mellitus	31.6 % (4367/13808)	25.1 % (8205/32649)	1.38 (1.32-1.44)	1.22 (1.17-1.28)
Renal insufficiency	21.5 % (2989/13888)	20.7 % (6807/32806)	1.05 (1.00-1.10)	0.73 (0.70-0.77)
On hemodialysis	1.9 % (257/13888)	2.0 % (643/32806)	0.94 (0.81-1.09)	0.83 (0.72-0.97)
Diagnostic XA and PCI in one session	95.3 % (13662/14336)	95.1 % (32230/33879)	1.04 (0.95-1.14)	1.14 (1.04-1.25)
- fluoroscopy time (min)*	8.9 (5.5; 14.0)	9.2 (5.9; 15.0)		
- contrast agent (ml)*	170 (130; 230)	180 (140; 248)		
PCI only	4.7 % (674/14336)	4.9 % (1649/33879)	0.96 (0.88-1.06)	0.88 (0.80-0.96)
- fluoroscopy time (min)*	8.9 (5.0; 15.0)	9.6 (5.5; 16.2)		
- contrast agent (ml)*	140 (100; 200)	150 (100; 205)		
PCI successful	94.6 % (13556/14336)	94.1 % (31893/33878)	1.08 (0.99-1.18)	1.17 (1.07-1.28)
Stent implanted	90.2 % (12935/14336)	89.8 % (30436/33879)	1.04 (0.98-1.12)	1.10 (1.02-1.17)
PCI in more than 1 vessel	11.9 % (1703/14336)	11.8 % (3987/33879)	1.01 (0.95-1.07)	0.93 (0.88-0.99)
PCI in complete vessel occlusion	17.4 % (2486/14288)	20.3 % (6836/33753)	0.83 (0.79-0.87)	0.92 (0.88-0.97)
PCI in CABG	3.0 % (429/14288)	6.8 % (2301/33753)	0.42 (0.38-0.47)	0.33 (0.30-0.37)
PCI in unprotected left main	1.8 % (252/14288)	1.6 % (551/33753)	1.08 (0.93-1.26)	0.88 (0.76-1.03)
PCI in ostial stenosis	7.0 % (1007/14288)	5.7 % (1910/33753)	1.26 (1.17-1.37)	1.13 (1.04-1.22)
PCI in last coronary vessel	0.5 % (73/14288)	0.5 % (179/33753)	0.96 (0.73-1.27)	0.83 (0.63-1.10)
Death in cath lab	0.2 % (28/14335)	0.1 % (39/33877)	1.70 (1.04-2.76)	1.38 (0.84-2.28)
TIA/stroke in cath lab	0.1 % (8/14335)	<0,1 % (14/33877)	1.35 (0.57-3.22)	1.00 (0.41-2.45)
Resuscitation in cath lab	0.6 % (88/14335)	0.3 % (101/33877)	2.07 (1.55-2.75)	1.78 (1.32-2.39)

Values are expressed as percentages (number of occurrences/available cases) unless indicated otherwise

*Median (interquartile range).

NSTE-ACS=non ST elevation acute coronary syndrome, OR=odds ratio, CI=confidence interval, XA=coronary angiography, PCI=percutaneous coronary intervention, CABG=coronary artery bypass graft.

Table S3. Base-line and procedural characteristics, PCI in STEMI, no cardiogenic shock

	Women<br>n=9156 (27,8 %)	Men<br>n=23830 (72,2 %)	Odds ratio<br>(95%-CI)	Age-adjusted<br>OR (95%-CI)
Age (years),*	72 (61; 79)	62 (53; 71)		
Prior XA	17.8 % (1579/8886)	23.1 % (5358/23175)	0.72 (0.68-0.76)	0.61 (0.57-0.65)
Prior PCI	12.4 % (1103/8875)	17.6 % (4057/23112)	0.67 (0.62-0.72)	0.59 (0.55-0.64)
Prior CABG	2.8 % (251/9028)	4.5 % (1065/23562)	0.60 (0.53-0.69)	0.42 (0.37-0.49)
Ejection fraction ≤40%	19.7 % (1161/5894)	18.6 % (2849/15304)	1.07 (0.99-1.16)	0.92 (0.85-0.99)
Diabetes mellitus	25.5 % (2115/8282)	17.7 % (3811/21547)	1.60 (1.50-1.70)	1.31 (1.23-1.40)
Renal insufficiency	16.4 % (1305/7934)	12.3 % (2550/20690)	1.40 (1.30-1.51)	0.87 (0.81-0.94)
On hemodialysis	1.0 % (81/7934)	0.8 % (164/20690)	1.29 (0.99-1.69)	0.99 (0.75-1.31)
Diagnostic XA and PCI in one session	98.1 % (8978/9156)	98.1 % (23372/23830)	0.99 (0.83-1.18)	1.08 (0.90-1.30)
- fluoroscopy time (min)*	8.0 (5.1; 13.1)	8.1 (5.2; 13.3)		
- contrast agent (ml)*	170 (130; 230)	180 (130; 240)		
PCI only	1.9 % (178/9156)	1.9 % (458/23830)	1.01 (0.85-1.21)	0.92 (0.77-1.11)
- fluoroscopy time (min)*	8.9 (5.1; 15.2)	8.0 (5.1; 13.3)		
- contrast agent (ml)*	150 (100; 200)	150 (100; 200)		
PCI successful	93.5 % (8557/9156)	94.7 % (22568/23830)	0.80 (0.72-0.88)	0.96 (0.87-1.07)
Stent implanted	91.1 % (8341/9156)	92.4 % (22028/23830)	0.84 (0.77-0.91)	0.96 (0.87-1.05)
PCI in more than 1 vessel	8.2 % (753/9155)	7.7 % (1825/23829)	1.08 (0.99-1.18)	0.98 (0.89-1.07)
PCI in complete vessel occlusion	52.5 % (4804/9146)	54.2 % (12915/23818)	0.93 (0.89-0.98)	0.98 (0.94-1.03)
PCI in CABG	1.1 % (103/9146)	2.2 % (517/23818)	0.51 (0.41-0.64)	0.33 (0.27-0.42)
PCI in unprotected left main	1.2 % (107/9146)	0.9 % (205/23818)	1.36 (1.08-1.72)	1.03 (0.80-1.31)
PCI in ostial stenosis	3.9 % (361/9146)	3.3 % (779/23818)	1.22 (1.07-1.38)	1.09 (0.96-1.25)
PCI in last coronary vessel	0.6 % (53/9146)	0.4 % (104/23818)	1.33 (0.95-1.85)	1.02 (0.72-1.44)
Death in cath lab	0.4 % (40/9156)	0.3 % (61/23830)	1.71 (1.15-2.55)	1.16 (0.76-1.76)
TIA/stroke in cath lab	0.1 % (5/9156)	<0.1 % (3/23830)	4.34 (1.04-18.16)	4.94 (1.12-21.72)
Resuscitation in cath lab	1.4 % (127/9156)	1.0 % (231/23830)	1.44 (1.16-1.79)	1.32 (1.06-1.66)

Values are expressed as percentages (number of occurrences/available cases) unless indicated otherwise

*Median (interquartile range).

STEMI=ST elevation myocardial infarction, OR=odds ratio, CI=confidence interval, XA=coronary angiography, PCI=percutaneous coronary intervention, CABG=coronary artery bypass graft.

Table S4. Base-line and procedural characteristics, PCI in cardiogenic shock

	Women<br>n= 963 (30,3 %)	Men<br>n= 2219 (69,7 %)	Odds ratio<br>(95%-CI)	Age-adjusted<br>OR (95%-CI)
Age (years)*	74 (66; 81)	67 (57; 75)		
Prior XA	25.7 % (221/860)	35.1 % (692/1969)	0.64 (0.53-0.76)	0.59 (0.49-0.70)
Prior PCI	17.8 % (154/864)	25.6 % (498/1943)	0.63 (0.51-0.77)	0.59 (0.48-0.72)
Prior CABG	7.0 % (64/919)	10.2 % (218/2145)	0.66 (0.50-0.88)	0.55 (0.41-0.75)
Ejection fraction ≤40%	62.4 % (406/651)	66.9 % (984/1471)	0.82 (0.68-0.99)	0.77 (0.63-0.94)
Diabetes mellitus	35.4 % (274/775)	31.4 % (544/1735)	1.20 (1.00-1.43)	1.00 (0.83-1.20)
Renal insufficiency	37.5 % (291/776)	36.6 % (654/1788)	1.04 (0.87-1.24)	0.76 (0.63-0.92)
On hemodialysis	4.5 % (35/776)	3.9 % (70/1788)	1.16 (0.77-1.76)	1.06 (0.69-1.63)
STEMI	77.6 % (747/963)	73.7 % (16536/2219)	1.23 (1.03-1.47)	1.45 (1.21-1.75)
Diagnostic XA and PCI in one session	97.9 % (943/963)	98.0 % (2175/2219)	0.95 (0.56-1.63)	0.99 (0.57-1.72)
- fluoroscopy time (min)*	10.1 (6.2; 15.6)	10.1 (6.0; 16.6)		
- contrast agent (ml)*	180 (120; 240)	185 (130; 250)		
PCI only	2.1 % (20/963)	2.0 % (44/2219)	1.05 (0.61-1.79)	1.01 (0.58-1.75)
- fluoroscopy time (min)*	10.9 (5.8; 16.6)	10.0 (6.0; 22.1)		
- contrast agent (ml)*	180 (110; 255)	195 (115; 285)		
PCI successful	86.1 % (829/963)	85.7 % (1901/2219)	1.03 (0.83-1.29)	1.19 (0.95-1.49)
Stent implanted	86.1 % (829/963)	85.9 % (1907/2219)	1.01 (0.81-1.26)	1.13 (0.90-1.41)
PCI in more than 1 area	21.0 % (202/963)	22.3 % (495/2219)	0.92 (0.77-1.11)	0.89 (0.74-1.08)
PCI in complete vessel occlusion	65.3 % (628/962)	62.0 % (1376/2218)	1.15 (0.98-1.35)	1.29 (1.09-1.51)
PCI in CABG	2.5 % (24/962)	4.7 % (105/2218)	0.51 (0.33-0.81)	0.41 (0.26-0.64)
PCI in unprotected left main	8.0 % (77/962)	9.1 % (202/2218)	0.87 (0.66-1.14)	0.80 (0.60-1.06)
PCI in ostial stenosis	11.1 % (107/962)	12.4 % (275/2218)	0.88 (0.70-1.12)	0.86 (0.67-1.09)
PCI in last coronary vessel	2.8 % (27/962)	4.3 % (96/2218)	0.64 (0.41-0.99)	0.62 (0.39-0.96)
Death in cath lab	7.4 % (71/963)	6.2 % (138/2218)	1.20 (0.89-1.61)	1.10 (0.81-1.49)
TIA/stroke in cath lab	0.2 % (2/963)	<0.1 % (1/2219)	4.62 (0.42-50.97)	3.26 (0.27-39.20)
Resuscitation in cath lab	14.4 % (139/963)	12.5 % (278/2219)	1.18 (0.95-1.47)	1.12 (0.89-1.40)

Values are expressed as percentages (number of occurrences/available cases) unless indicated otherwise

*Median (interquartile range).

PCI=percutaneous coronary intervention, OR=odds ratio, CI=confidence interval, XA=coronary angiography, CABG=coronary artery bypass graft, STEMI=ST elevation myocardial infarction.

Table S5. In-Hospital course and procedure-related complications, elective PCI

	Women<br>n=24262 (26,9 %)	Men<br>n=65972 (73,1 %)	Odds ratio<br>(95%-CI)	Age-adjusted<br>OR (95%-CI)
Hospital death	0.3 % (82/24262)	0.2 % (154/65972)	1.45 (1.11-1.90)	1.07 (0.82-1.41)
Non-fatal myocardial infarction	0.3 % (80/24119)	0.2 % (118/65810)	1.85 (1.39-2.46)	1.80 (1.35-2.41)
Non-fatal TIA/stroke	0.1 % (23/24262)	0.1 % (51/65972)	1.23 (0.75-2.01)	1.03 (0.63-1.70)
Hospital MACE	0.7 % (162/24262)	0.4 % (272/65972)	1.62 (1.34-1.97)	1.37 (1.12-1.67)
Hospital MACCE	0.8 % (184/24262)	0.5 % (323/65972)	1.55 (1.30-1.86)	1.31 (1.09-1.57)
Non-fatal pulmonary embolism	<0.1 % (1/24123)	<0.1 % (1/65819)	2.73 (0.17-43.62)	3.09 (0.18-52.07)
Non-fatal resuscitation	0.2 % (44/24262)	0.1 % (90/65972)	1.33 (0.93-1.91)	1.33 (0.92-1.91)
Access-related complications	1.8 % (447/24245)	0.8 % (553/65932)	2.22 (1.96-2.52)	2.07 (1.83-2.36)
Other non-fatal complications	0.9 % (226/24262)	0.7 % (494/65972)	1.25 (1.06-1.46)	1.23 (1.04-1.44)
Emergency CABG or	0.2 % (41/18813)	0.2 % (85/50896)	1.31 (0.90-1.90)	1.31 (0.90-1.92)
Hospital stay (days)	3 (1; 6)	2 (1; 5)		

Values are expressed as percentages (number of occurrences/available cases) except for hospital stay (median; interquartile range).

PCI=percutaneous coronary intervention, OR=odds ratio, CI=confidence interval, TIA=transient ischemic attack, MACE=major adverse cardiac event (death, non-fatal myocardial infarction), MACCE=major adverse cardiac and cerebrovascular event (death, non-fatal myocardial infarction, hospital TIA, and hospital stroke), CABG=coronary artery bypass graft

Table S6. Hospital course and procedure-related complications, PCI in NSTE-ACS, no cardiogenic shock

	Women<br>n= 14336 (29,7 %)	Men<br>n= 33879 (70,3 %)	Odds ratio<br>(95%-CI)	Age-adjusted<br>OR (95%-CI)
Hospital death	2.4 % (350/14336)	1.8 % (597/33879)	1.40 (1.22-1.59)	1.02 (0.89-1.16)
Non-fatal myocardial infarction	0.3 % (38/14224)	0.2 % (73/33755)	1.24 (0.83-1.83)	1.24 (0.83-1.86)
Non-fatal TIA/stroke	0.2 % (27/14336)	0.1 % (34/33879)	1.88 (1.13-3.11)	1.57 (0.94-2.65)
Hospital MACE	2.7 % (388/14336)	2.0 % (670/33879)	1.38 (1.21-1.56)	1.04 (0.91-1.18)
Hospital MACCE	2.9 % (415/14336)	2.1 % (704/33879)	1.40 (1.24-1.59)	1.06 (0.94-1.21)
Non-fatal pulmonary embolism	<0.1 % (2/14232)	<0.1 % (1/33762)	4.75 (0.43-52.33)	4.11 (0.35-48.44)
Non-fatal resuscitation	0.3 % (47/14336)	0.2 % (67/33879)	1.66 (1.14-2.41)	1.61 (1.10-2.37)
Access-related complications	2.3 % (324/14299)	1.0 % (352/33844)	2.21 (1.89-2.57)	1.99 (1.70-2.33)
Other non-fatal complications	1.5 % (218/14336)	1.1 % (359/33879)	1.44 (1.22-1.71)	1.36 (1.14-1.62)
Emergency CABG	0.3 % (37/10958)	0.3 % (65/25770)	1.34 (0.89-2.01)	1.33 (0.88-2.01)
Hospital stay (days)	6 (3; 10)	5 (2; 8)		

Values are expressed as percentages (number of occurrences/available cases) except for hospital stay (median; interquartile range).

PCI=percutaneous coronary intervention, NSTE-ACS=non ST elevation acute coronary syndrome, OR=odds ratio, CI=confidence interval, TIA=transient ischemic attack, MACE=major adverse cardiac event (death, non-fatal myocardial infarction), MACCE=major adverse cardiac and cerebrovascular event (death, non-fatal myocardial infarction, hospital TIA, and hospital stroke), CABG=coronary artery bypass graft

Table S7. Hospital course and procedure-related complications, PCI in STEMI, no cardiogenic shock

	Women n= 9156 (27,8 %)	Men n= 23830 (72,2 %)	Odds ratio (95%-CI)	Age-adjusted OR (95%-CI)
Hospital death	6.3 % (581/9156)	3.6 % (854/23830)	1.82 (1.64-2.03)	1.19 (1.06-1.33)
Non-fatal myocardial infarction	0.3 % (27/9114)	0.2 % (52/23761)	1.35 (0.85-2.16)	1.25 (0.77-2.03)
Non-fatal TIA/stroke	0.1 % (13/9156)	0.1 % (18/23830)	1.88 (0.92-3.84)	1.59 (0.75-3.36)
Hospital MACE	6.6 % (608/9156)	3.8 % (906/23830)	1.80 (1.62-2.00)	1.19 (1.07-1.34)
Hospital MACCE	6.8 % (621/9156)	3.9 % (924/23830)	1.80 (1.62-2.00)	1.20 (1.08-1.34)
Non-fatal pulmonary embolism	0,0 % (1/9115)	0,0 % (0/23764)	--	--
Non-fatal resuscitation	0.7 % (65/9156)	0.6 % (154/23830)	1.10 (0.82-1.47)	1.18 (0.87-1.59)
Access-related complications	2.2 % (201/9142)	0.8 % (201/23808)	2.64 (2.17-3.22)	2.32 (1.89-2.85)
Other non-fatal complications	1.5 % (138/9156)	1.2 % (291/23830)	1.24 (1.01-1.52)	1.15 (0.93-1.43)
Emergency CABG	0.4 % (28/6920)	0.3 % (57/18407)	1.31 (0.83-2.06)	1.22 (0.76-1.95)
Hospital stay (days)	7 (3; 10)	7 (3; 9)		

Values are expressed as percentages (number of occurrences/available cases) except for hospital stay (median; interquartile range).

PCI=percutaneous coronary intervention, STEMI=ST elevation myocardial infarction, OR=odds ratio, CI=confidence interval, TIA=transient ischemic attack, MACE=major adverse cardiac event (death, non-fatal myocardial infarction), MACCE=major adverse cardiac and cerebrovascular event (death, non-fatal myocardial infarction, hospital TIA, and hospital stroke), CABG=coronary artery bypass graft

Table S8. Hospital course and procedure-related complications, PCI in cardiogenic shock

	Women n= 963 (30,3 %)	Men n= 2219 (69,7 %)	Odds ratio (95%-CI)	Age-adjusted OR (95%-CI)
Hospital death	46.5 % (448/963)	40.4 % (896/2219)	1.28 (1.10-1.50)	1.05 (0.89-1.23)
Non-fatal myocardial infarction	0.1 % (1/958)	0.3 % (6/2204)	0.38 (0.05-3.18)	0.48 (0.06-4.09)
Non-fatal TIA/stroke	0.0 % (0/963)	<0.1 % (1/2219)	--	--
Hospital MACE*	46.6 % (449/963)	40.6 % (902/2219)	1.28 (1.10-1.49)	1.04 (0.89-1.22)
Hospital MACCE†	46.6 % (449/963)	40.7 % (903/2219)	1.27 (1.09-1.48)	1.04 (0.89-1.22)
Non-fatal pulmonary embolism	0.0 % (0/958)	0.0 % (0/2205)	--	--
Non-fatal resuscitation	2.7 % (26/963)	3.2 % (70/2219)	0.85 (0.54-1.34)	0.93 (0.59-1.49)
Access-related complications	1.1 % (11/961)	0.4 % (9/2206)	2.83 (1.17-6.84)	2.66 (1.07-6.63)
Other non-fatal complications	1.8 % (17/963)	2.9 % (64/2219)	0.61 (0.35-1.04)	0.67 (0.39-1.16)
Emergency CABG	2.1 % (11/515)	1.1 % (14/1244)	1.92 (0.86-4.25)	2.09 (0.93-4.71)
Transfer to other hospital	9.1 % (88/962)	10.5 % (233/2219)	0.86 (0.66-1.11)	0.95 (0.73-1.24)
Hospital stay (days)	6 (1; 14)	8 (1; 16)		

Values are expressed as percentages (number of occurrences/available cases) except for hospital stay (median; interquartile range).

***** death, non-fatal myocardial infarction

† death, non-fatal myocardial infarction, stroke

PCI=percutaneous coronary intervention, OR=odds ratio, CI=confidence interval, TIA=transient ischemic attack, MACE=major adverse cardiac event (death, non-fatal myocardial infarction), MACCE=major adverse cardiac and cerebrovascular event (death, non-fatal myocardial infarction, hospital TIA, and hospital stroke), CABG=coronary artery bypass graft
